# Anisotropic Microstructure and Performance Characterization of Wild Silkworm Cocoons for Designing Biomimetic Protective Materials

**DOI:** 10.3390/polym14153072

**Published:** 2022-07-29

**Authors:** Mengru Li, Jie Luo, Yi Xiong, Jisong Wu

**Affiliations:** 1 School of Fine Arts & Design, Guangzhou University, Guangzhou 510006, China; mengru.li@gzhu.edu.cn (M.L.); msxy@gzhu.edu.cn (Y.X.); 2 School of Textile Science and Engineering, Wuhan Textile University, Wuhan 430200, China; jisongwu11@163.com

**Keywords:** silkworm cocoon, mechanical properties, composites, structure-properties, biomimetic protective materials

## Abstract

As a unique and important biopolymer composite, silkworm cocoons have evolved a wide range of different structures and combinations of physical and chemical properties to resist environmental damage and attacks from natural predators. A combination of characterization techniques including scanning electron microscopy, mechanical tests, and Fourier transform infrared spectroscopy were applied to investigate the morphologies, mechanical properties, and nanoscale organizations of Antheraea pernyi cocoons from two different source regions. Mechanical tests were carried out by using rectangular specimens cut from four directions 0° (width of the cocoons), ±45°, and 90° (the length of the cocoon), separately. The mechanical properties such as tensile strength, initial modulus, and maximum load of cocoon in four directions were measured. The structural analysis of silkworm cocoon shows that there is a slightly different combination of morphology and properties that have adapted to coping with diverse local environments. The results of the mechanical properties of silkworm cocoons show that the A. pernyi cocoon from north of China behaved stronger and tougher. Besides, there were slight differences among the results of mechanical properties for 0°, ±45°, and 90° directions of these cocoons. Our studies will help formulate bio-inspired design principles for new materials.

## 1. Introduction

**Figure 1:**
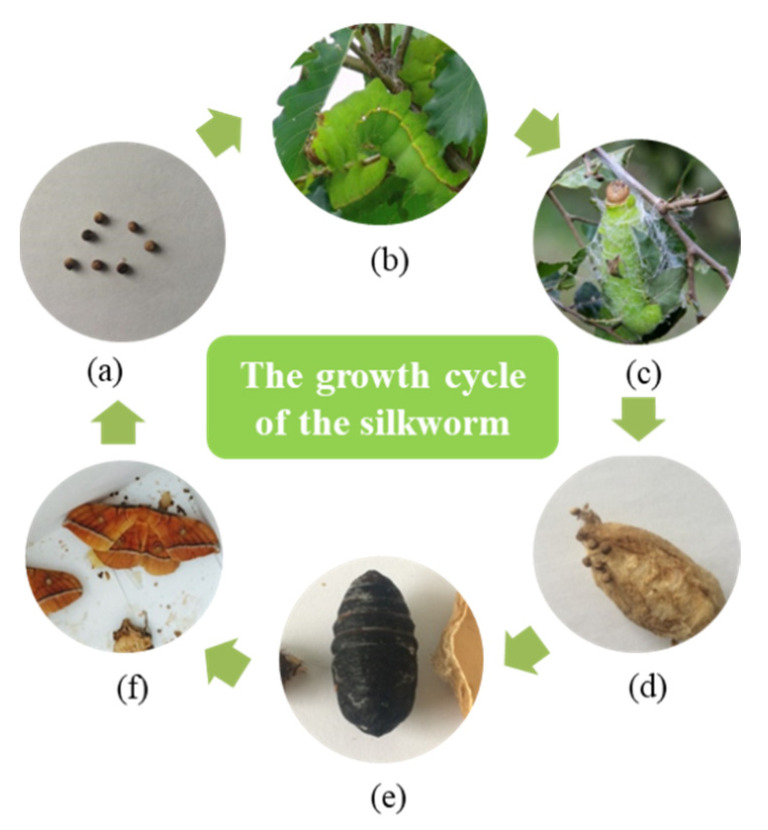
The growth cycle of *A. pernyi*: (**a**) eggs laid by an adult moth; (**b**) larva; (**c**) silkworm spinning; (**d**) cocooning; (**e**) preadult; (**f**) an adult moth.

Cocoon is a type of unique and important biopolymer composite in nature with excellent microstructure and ecological functions, which plays important roles in the transformation from silkworm and pupa to adult moth. In comparison with domesticated silkworm, such as *Bombyx mori* (*B. mori*), wild silkworm cocoons, such as *Antheraea pernyi* (*A. pernyi*), are reared in the open environment require much greater protection from environmental, biotic and physical hazards [1]. The *A. pernyi* cocoon is one of the major sources of wild silk fibers [2]. The growth of the silkworm cocoon undergoes several stages: ovum, larva (feeding phase), silkworm spinning, cocooning, preadult, and silkworm moth, as shown in Figure 1. After hundreds of millions of years evolution, the *A. pernyi* can produce cocoons with special structure and functions, which can provide a suitable temperature, humidity, and living environment for silkworm pupae in the incubation process, and avoid external damage such as wasp stings and bird pecking [3]. Guo et al. [4] studied the gradient structure and property of cocoon layers, which could provide inspiration for the separator design research focusing on the high safety and high performance for the development of lithium-ion batteries. Kwak et al. [5] developed high-toughness natural polymer nonwoven preforms inspired by silkworm cocoon structure with excellent mechanical strength and high physical stability. Hu et al. [6] provided a new way to design better static puncture resistant materials by studying the structure of cocoons. Therefore, studying the internal microstructure of silkworm cocoons is beneficial to the further application of these natural biopolymer composites and developing the biomimetic materials.

Nowadays, much attention has been given to the field of mechanical properties of silkworm cocoons for years [1,7]. Despite the rapid growth of research interest in *B. mori* cocoons [8,9,10,11], limited studies have been conducted to understand the structure and functions of wild silkworm cocoons. For example, Zhou et al. [12] studied the structural characteristics and differences in performance of every layer of the Eri Silkworm cocoon as well as their role in the cocoon’s mechanical protection, humidity control, temperature buffering, and UV protection. Zhou et al. [13] compared the morphological structure and basic properties of stereoscopic cocoons, flat cocoons, and multi-silkworm flat cocoons, which were constructed by *B. mori* from different cocooning sites for single or multiple silkworms. Moreover, some research also carried out a comparative study of domestic silkworm and wild silkworm. Chen et al. [14] described a diversity of structural features of 27 different species of silkworm cocoons. Zhang et al. [1] explored the structure and mechanical property relationships of four types of silkworm cocoon walls (domesticated *B. mori*, semidomesticated Antheraea assamensis, and wild *A. pernyi* and Antheraea mylitta silkworm cocoons) by peeling, out-of-plane compression, and nano-indentation tests and analysis of microstructure. Guan et al. [15] showed that *B. mori* behaves as a weak and brittle fiber composite, while *A. pernyi* behaved strong and tough. Song et al. [16] studied the microstructure of domesticated and wild silkworm cocoons by using X-ray micro computed tomography.

In recent years, much attention has been given to non-mulberry cocoon types such as *A. pernyi*, because of its special mechanics, thermal regulation [17,18,19], puncture resistance [20], and UV screening properties [21,22]. In order to maximize the utilization of green resources and to produce silk materials that are suitable for different applications, it is necessary to understand the structure and properties of fibers from different components of a wild cocoon. Studies conducted on the *A. pernyi* cocoon in the past few years were also limited to the cocoon shell and fibers within the shell [1]. For instance, Du et al. [23] studied the silk fibers from three key components of the *A. pernyi* silkworm, i.e., peduncle, outer floss, and the cocoon shell (both outermost and pelade parts). Dai et al. [24] conducted a comparative analysis of iTRAQ-based proteomes for cocoons between the domestic silkworm (*B. mori*) and wild silkworm (Bombyx mandarina). However, limited research has recently been conducted on the performance and function of *A. pernyi* cocoons from different source regions.

Therefore, the main aim of this study is to explore the effects of different environmental characteristics on the microscopic and macroscopic morphology, composition, and the mechanical properties of *A. pernyi* cocoons by scientific experimental methods, and to obtain a clear understanding of the structure-property of silk materials, structural characterization and analysis at a molecular level is required. Two different *A. pernyi* cocoons from two source regions were selected as experimental objects. The specifications, surface morphology, and section microstructure were observed and compared. Besides, the complete stress-strain curves of rectangular specimens cut from these cocoons in different directions (0°, ±45°, and 90°) were discussed and the relationship between the microstructure and properties were also analyzed. The microstructure and the fracture surfaces of cocoons were observed by scanning electron microscopy (SEM). Exploration of the physical and mechanical properties of this kind of natural polymer composite materials might provide inspirations for designing and developing the next-generation bio-mimic protective materials.

## 2. Materials and Methods

### 2.1. Preparation of Materials

The *A. pernyi* cocoons from two source regions were selected and the source region of these cocoons were the Henan province (*A. pernyi* H, Middle-China) and the Liaoning province (*A. pernyi* L, North China), separately. In China, north latitude 36° is the demarcation line of *A. pernyi* silkworm’ voltinism, as the *A. pernyi* silkworm in the area of the south of demarcation line is classified as univolitine and the *A. pernyi* silkworm in the area of north of demarcation line is classified as bivoltine races. The geographical coordinates of Nanyang (in the Henan province) and Dandong (in the Liaoning province), are 33°and 40°07′ north latitude, respectively. Therefore, the *A. pernyi* silkworm cocoons used in this study are univolitine (*A. pernyi* (H)) and bivoltine races (*A. pernyi* (L)). The two kinds of *A. pernyi* silkworm cocoons were free-ranging in the mountains and mainly eating tussah leaves. Besides, the time of harvest is at the beginning of September. All the cocoons were stored in the fresh-keeping area of the refrigerator under the same environmental conditions to prevent the live pupas from hatching. In addition, the pupa inside were removed from these silkworm cocoons before the test. Biological replication has been carried out for all the tests in this study. The cocoons were measured or tested by random selection. Potassium bromide and anhydrous ethanol analytical pure (AR) were purchased from Sinopharm Chemical Reagents Co., LTD, Shanghai, China.

### 2.2. Specifications Measurements

**Figure 2:**
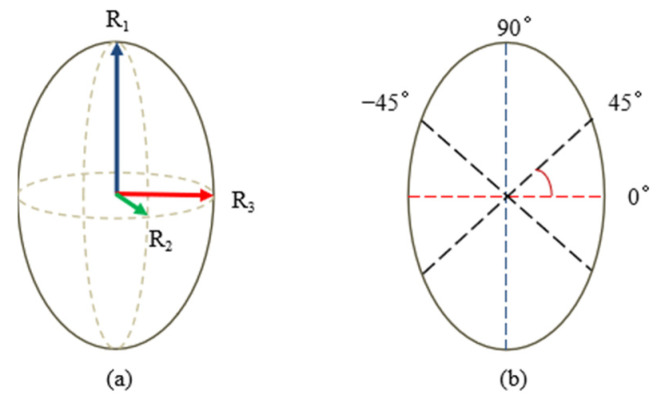
The ellipsoidal model of the *A. pernyi* cocoon (**a**) three-dimensional ellipsoidal model; (**b**) areal ellipsoidal model.

The elliptical model of cocoon was shown in Figure 2. Ten samples of cocoons from different source regions were randomly selected and we weighed the cocoons without the pupa. The physical dimensions of the *A. pernyi* cocoon, including the length of the cocoon (2R_1_), width of the cocoons in minor (2R_2_), and major axes (2R_3_), were measured by using an electronic vernier caliper (Guanglu Co., LTD, Dongguan, China). Besides, the thickness of the cocoon layer was measured using an electronic vernier caliper by cutting the cocoon with special tools. Each sample was measured 5 times for the average value.

### 2.3. Scanning Electron Microscope (SEM) Observation

The cocoon layers with a damaged puncture area were cut into strips with a dimension of 3 mm × 3 mm, which were then attached to conductive tape on aluminum stubs. The microstructure of *A. pernyi* cocoon was observed by Scanning Electron Microscope (SEM) (JSM6510, JEOL, Tokyo, Japan) under constant temperature and humidity (20 °C, 65% humidity) after sputtering with gold for 70 s. The fiber bonding length, which can be roughly linked to the fiber diameter, of the silk from different cocoon layers (outer layer, inter layer, and inner layer) was also measured by using Image J software. The Image J software was used to analyze a SEM image to find the porosity [25].

### 2.4. Fourier Transform Infrared Spectra (FITR)

The silk of two *A. pernyi* cocoons was cut into uniform fine powders and mixed evenly with dried potassium bromide (A.R.), respectively, in the agate mortar under a constant temperature and humidity environment of 20 °C and 65%. These two mixed powders were loaded into the mold and pressed into slices. The test was carried out by a NEXUS470 Fourier infrared spectrometer (Thermo Nicolet Corporation, Waltham, MA, USA) with the blank KBr tablet as the comparison test. Data was collected from 300 to 4000 cm^−1^ with a nominal resolution of 4 cm^−1^ and scanned 32 times.

### 2.5. Tensile Properties Test

To test the tensile strength, the *A. pernyi* cocoon layer was cut into 15 mm × 5 mm splines along four directions 0° (the width of the cocoon), the ±45°, and 90° (the length of the cocoon) separately, as shown in Figure 2b. The test samples were put into the laboratory for 48 h before tensile properties test. The tests were carried out using an Instron 5967 with speed of 2 mm/mm and 5 mm gauge length.

## 3. Results and Discussions

### 3.1. Specifications of Cocoon

**Figure 3:**
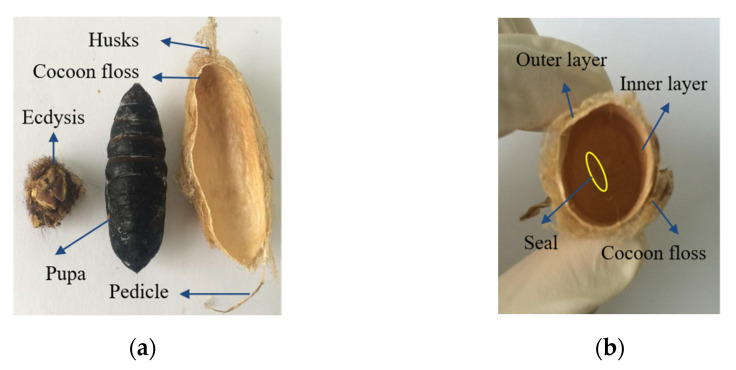
Digital images of the *A. pernyi* cocoons (**a**) vertical section; (**b**) cross section.

Figure 3 shows the photographic images of the *A. pernyi* cocoon and a cocoon shell of different layers. The porous *A. pernyi* cocoon consists of five parts: pedicle, husks, pupa, cocoon floss, and ecdysis, which morphologically has extra cocoon grip or peduncle and minerals by compared to the *B. mori* cocoon [7]. The cocoons from two source regions are mostly long oval. The lengths of the cocoon handle, which is the unique component of *A. pernyi* cocoons, are different. As shown in Figure 3a, the upper part is long-pointed, the middle part is wider, and the lower part is slightly blunt and relatively soft. Besides, on visual observation, the surface of the inner layer cocoon was found to be extremely smooth compared to the surface of the outer layer cocoon. In contrast, there is a conical, closed, and invisible hole in the lower part, that is, the sealing part, which is also the unique structure of the tussah cocoon, as shown in Figure 3b.

**Figure 4:**
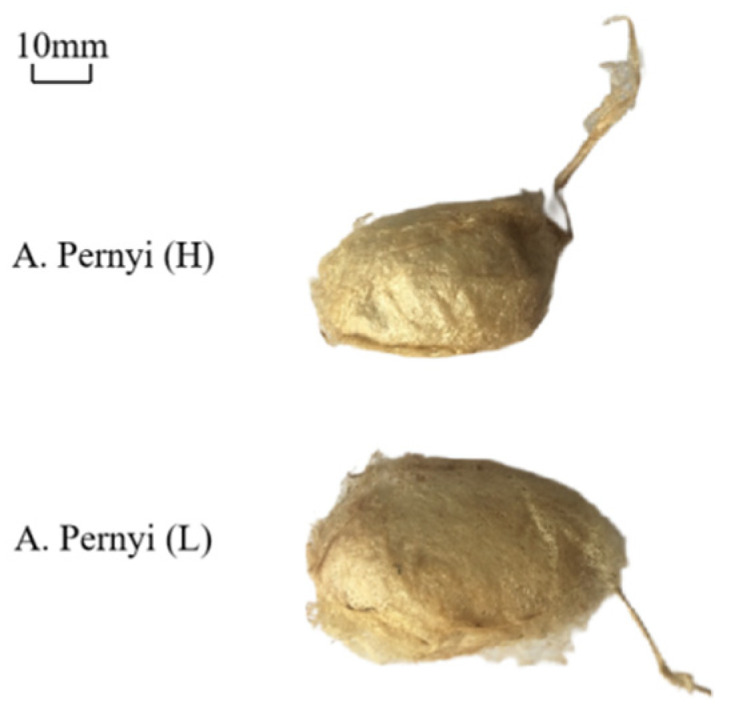
The comparison of the cocoons’ morphology.

Cocoons vary in weight, thickness, color, and stiffness due to the rearing environment [1]. The geometrical parameters of *A. pernyi* cocoons were measured and summarized in Table 1. By comparison, the weight, thickness and the size of *A. pernyi* (L) and *A. pernyi* (H) have a significant difference (*p*-value < 0.01), as the former is higher and larger than the corresponding part of the latter, as shown in Figure 4 and Table 1. The thin wall is less than 1 mm in thickness. The aspect ratio of an ellipsoidal cocoon, defined as the length ratio between the long- and the short-axes, is around 1.84~1.95. Both these two silkworm cocoons have brown-yellow pigment as the protective color [26]. The outer layer appears flossy due to the relatively weak interlayer bonding for forming a three-dimensional non-woven structure in the cocoon [3]. There are many uneven wrinkles on the outer surface of the cocoon [27], which is gradually formed during the process of spinning silk into cocoons. The reason is that the outer silk of *A. pernyi* was spun first, and therefore was dried faster and shrunk more because the sun shined on it, as shown in Figure 4. The inner layer was spun later, which then dried slowly with less shrinkage and was relatively compact and smooth, as shown in Figure 3b. The structural and morphological type of cocoons employed is usually constant within a genus [14].

### 3.2. Morphological Characteristics of the Component Layers of Silkworm Cocoon

**Figure 5:**
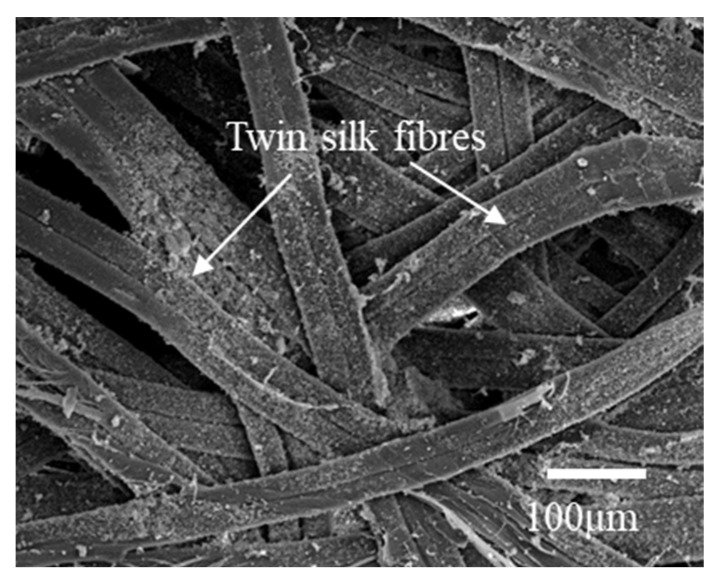
The non-woven cocoon structure from the *A. pernyi*.

It can be seen from the SEM image of Figure 5 that the silk consists of two threads bonded by sericin, which is a group of glue proteins spun by the middle silk gland of the silkworm. Fibroin fibers were surrounded by sericin and were fixed to each other in the cocoons. At the most general level, the *A. pernyi* cocoon is an optimal and a multi-layer porous fiber structure made of overlaying bave and a limited amount of raw materials. Both layers may be considered as a porous matrix of sericin reinforced by randomly oriented continuous fibroin fibers [9]. The silkworm cocoon comprises multiple layers along with high porosity, which is attributed to a cluttered nonwoven structure [3,14,28]. Silk sericin in the percentage of 30–35% of the whole cocoon [5,29], as a natural binder, provides the inter fiber and inter layer adhesion to form a structural integrity composite cocoon and enhance the mechanical properties of cocoons. *A. pernyi* silk fiber is wide and flat, and each silk is formed by continuous twin silk filaments bonded by sericin [3].

**Figure 6:**
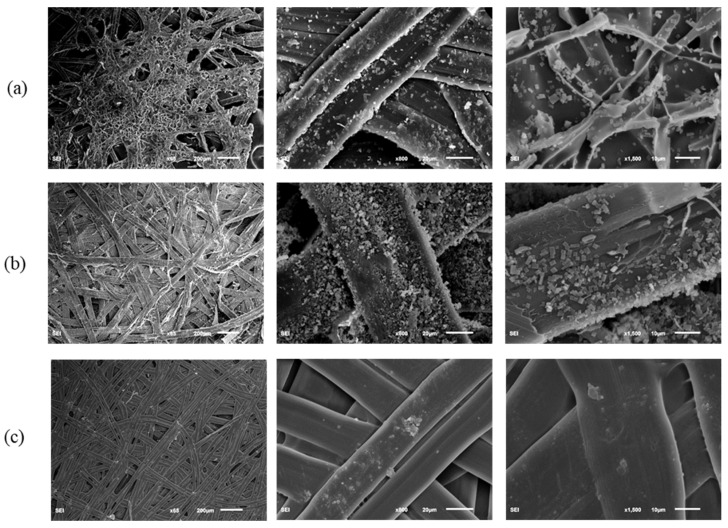
The micro-structure of different cocoon layers from *A. pernyi* (H): (**a**) outer layer, (**b**) middle layer, (**c**) inner layer.

**Figure 7:**
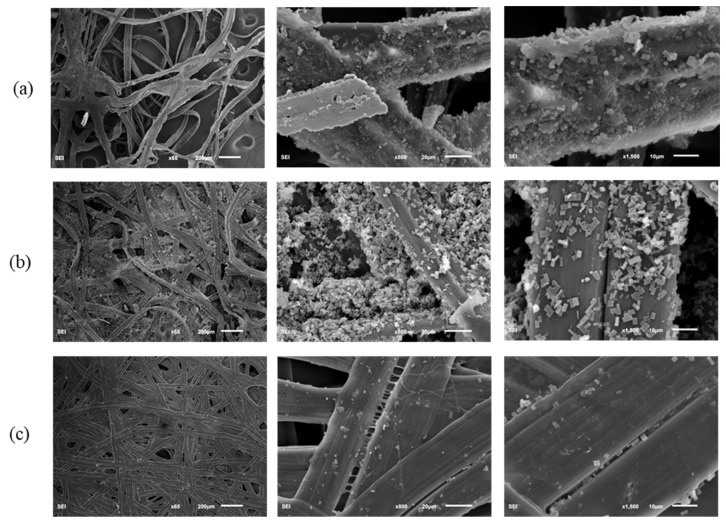
The micro-structure of different cocoon layers from *A. pernyi* (L): (**a**) outer layer, (**b**) middle layer, (**c**) inner layer.

**Figure 8:**
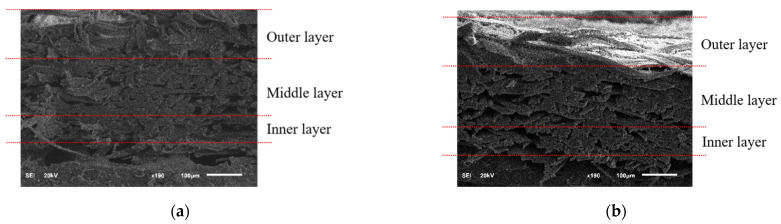
The cross section of the cocoon: (**a**) *A. pernyi* (H), (**b**) *A. pernyi* (L).

The SEM images in Figure 6 and Figure 7 show the micro-structures of different *A. pernyi* cocoon layers (outer layer, middle layer, and inner layer) from two source regions at different magnifications to make a comparison on their morphologies. Cocoons with this structure always have a graded layer structure, with the porosity decreasing through the thickness direction from the outer layer to the inner layer [14]. Compared with the cocoon of the middle and the inner layers, the pores in the cocoon of the outer layer are more numerous and larger. The outer silk is loose and round, while the inner silk is interlaced tightly and is smooth and flat, as can be seen in Figure 8. It is seen in Figure 6 and Figure 7 that the inner layer has a lower porosity (i.e., a higher silk density), which can be certified by the results shown in Table 2.

Besides, the cocoon surface is not smooth and was loosely stacked to dense and cubic crystals with different size, uneven shape, and un-uniform distribution. These crystals, identified as calcium oxalates [14], are deposited on the outer and middle layer surface of the outer layer fiber [6]. This feature may have a functional role, such as preferential gating of CO_2_ from the cocoon inside to outside and temperature regulation to maintain a physiological temperature inside the cocoon irrespective of the surrounding environment [30]. These varied size crystals are piled up on the silk fibers, especially in the crevices where fibers cross, and filling the gaps between them, thereby decreasing the cocoon porosity, as shown in Figure 6b and Figure 7b. They did not show any influence in enhancing the interlaminar adhesion between the cocoon layers, but exhibited much higher hardness than the cocoon pelades [1]. They contribute by trapping still air inside the cocoon structure and enhancing the thermal stability of the cocoon [16]. The wider function of calcium oxalate has not yet been investigated in detail and this trait, shared by many cocoons, is hence still little understood [30]. Besides, little crystals were observed on the surface of the inner layer cocoon, as shown in Figure 6c and Figure 7c.

Moreover, the fiber bonding length from the SEM pictures of cocoon has been roughly measured, which can be roughly linked to the fiber diameters (Table 2). From Table 2, it can be seen that the fiber bonding length (i.e., fiber diameter) of the silk from different layers of *A. pernyi* (L) is higher than the corresponding part of *A. pernyi* (H). Besides, the bonding length of outermost layer is a little larger than the innermost surface due to the faster spinning speed of silkworm cocoons. All of the parameters manifest like this may ascribe to the spinning method of cocoons [31]. In general, with the increase of spinning speed of silkworm cocoons, the fibers become finer and the fiber arrangement becomes denser. As the materials inside the silkworm caterpillar diminished, the movement speed of the caterpillar begins to undulate [32].

Comparisons were performed to find the differences of porosity between the outer layer, middle layer, and inner layer of the cocoons (Table 2). The results show that porosity has significant differences in the cocoons’ outer layer, middle layer, and inner layer. It can be seen that the outer and inner layers have the highest and lowest porosity in both the two *A. pernyi* cocoons. Besides, the porosity of *A. pernyi* (H) cocoon layers are larger than the corresponding part of *A. pernyi* (L) cocoon layers.

Moreover, the cross-sectional images of the *A. pernyi* (H) and *A. pernyi* (L) cocoons were shown in Figure 8. The cross section of the silk from the outer layer is flat and the arrangement is regular. The cross section of the middle layer is more regular than silk from the outer layer and the arrangement is relatively fluffy. The cross-section of silk from the inner layer is flat and the distribution is compact. Therefore, it can be noted that from the inner layer to the outer layer, the morphological structure of the fiber surface is first densely packed, then becomes loose and finally begins to be closely embraced.

### 3.3. FTIR Spectra Analysis

**Figure 9:**
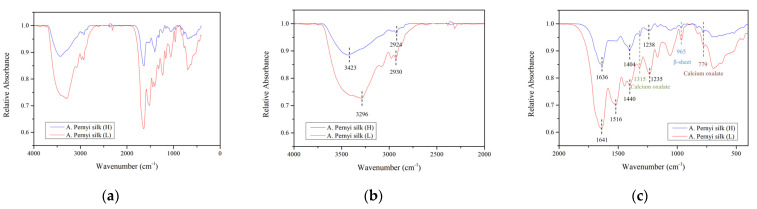
Comparison of FTIR of the *A. pernyi* cocoons (**a**) in the range 4000−400 cm^−1^; (**b**) in the range 4000−2000 cm^−1^; (**c**) in the range 2000−500 cm^−1^.

In Figure 9a, the FTIR spectra showed absorption peaks over the range 4000−400 cm^−1^ using 32 scans of the *A. pernyi* cocoons. The infrared spectrum of silk fiber is formed according to the different vibration wave bands generated by the structural characteristics of amide groups in the protein polypeptide chain of the fiber. The silk spectra are typical with a characteristically strong polypeptide backbone amide absorption bands. For proteins, the main group is amide, and all the amide bands can be characterized as a combination of separate contributions from the various protein structural motifs such as β-sheets, α-helices, turns, and random coils [33]. There are some characteristic peaks in Figure 9b, and the analysis of these characteristics can be found below. The higher wave number broad absorption at 3423 cm^−1^ as observed from the raw outer surface of *A. pernyi* cocoon (H) shows the presence of other functional groups in addition to the vibration arising out of O-H due to the presence of water in Figure 9a. In the absorption peaks of the 3300−2800 cm^−1^ region (C-N stretching vibration), 3300−3000 cm^−1^ and 3000−2800 cm^−1^ are unsaturated and saturated C-H stretching vibration absorption separately. The weak wave number broad absorption around 2927 cm^−1^ was observed vibration arising out of C-H.

For protein components, the most discriminating zone in FTIR spectra is the so-called fingerprint region, located around 1700−700 cm^−1^, where the complexity of the vibrational components is expressed [34]. It is well-known that the amide I bands (around 1700−1590 cm^−1^) and amide II bands (around 1590−1460 cm^−1^) are extremely sensitive to atmospheric water vapor and amide III bands (around 1280−1190 cm^−1^) is less-water sensitive [35]. Therefore, when analyzing the secondary structure of proteins, amide III is generally taken as the main research object. In Figure 9c, the very strong vibration around 1632 cm^−1^ (1636 cm^−1^ and 1641 cm^−1^, respectively) is assigned to asymmetric CO vibration and the strong vibration around 1315 cm^−1^ is assigned as symmetric CO vibration of the oxalate group [29]. The two main diagnostic bands identified for calcium oxalate hydrate are the O-CO out of phase bonding at 779 cm^−1^ and the asymmetric C=O stretching at 1315 cm^−1^ [14]. The two peaks corresponding to calcium oxalate at 1315 cm^−1^ and 779 cm^−1^ are well identified. Besides, the adsorption peaks show as 1240 cm^−1^ in amide III band and 695−700 cm^−1^ in amide V band, β-sheet. In the 960−966 cm^−1^ region, there is a moderate absorption (amide Ⅳ band, β-sheet). The study shows that alanine and alanine are connected to each other in the silk protein molecule, forming the propyl-propyl peptide chain structure, and the absorption peak of ~965 cm^−1^ is the characteristic absorption band of this structure and correspond to the C-N stretching and movements of CH_3_ groups and/or to the N-H rocking [36] from the Ala-Ala peptide structure constituting β-sheet crystals. It can be seen that the characteristic peaks of silk in the two regions are basically the same, that is, the protein secondary structure is very similar, but it is different, to some extent, to the absorption strength and the vibration strength of the wave of tussah silk in the two different regions.

Magoshi et al. [37] proposed to use the ratio of two bands ($\frac{RA_{1265}}{RA_{1235}}$) as the basis for the qualitative and quantitative determination of the crystallization index of the silk. As shown in Table 3, silk from *A. pernyi* (L) shows the higher crystallization index than the corresponding part of *A. pernyi* (H), which indicated that the silk in *A. pernyi* (L) has relatively higher oriented fiber.

### 3.4. Tensile Mechanical Properties of Cocoon Composites

**Figure 10:**
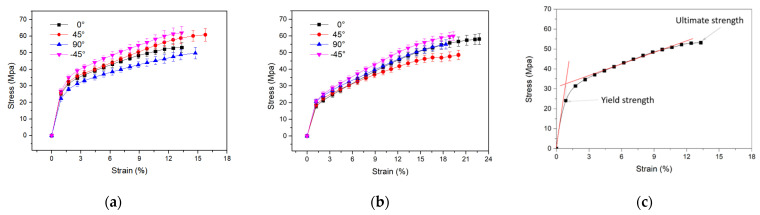
Typical tensile stress-strain curves of rectangular specimens cut from cocoon: (**a**) *A. pernyi* cocoon (H); (**b**) *A. pernyi* cocoon (L); (**c**) different stages in typical stress-strain curve of *A. pernyi* cocoon (H) in the 0° direction.

The tensile properties of samples were investigated in the cocoon of 0°, ±45°, and 90° directions. Several representative tensile stress-strain curves of rectangular specimens of the plane cocoon walls obtained from tension tests are given in Figure 10. It is observed that these two cocoons have a similar general form to their tensile stress–strain deformation profile in the plane of the cocoon wall. Although delaminations of the sub-layers occur in the thickness directions due to the relatively weak bonding of sericin of the outermost layer during tension, the stress rises nonlinearly with the increasing strain prior to failure, which has more complex stress-strain profiles and is correlated well with the large elongation of wild silk fibers [38]. The stress rises with strain to a maximum value and the gradient of these curves change twice through apparent yield points until the stress falls relatively rapidly after the maximum strain. Then, the cocoon shell specimen enters its “plastic” stage, where a post-yield modulus can be obtained (Table 3). The bonding of sericin was damaged, as a failed planar structure was observed after tensile failure from *A. pernyi* due to the stronger bond in the 3D cocoon fibrous assembly [39]. Then, the fibers were broken, which is the main fracture mechanism for the cocoons [17].

In addition, the *A. pernyi* cocoon (L) exhibits a high ability of elastic deformation, with an elastic limit strain higher than 18% in the four directions. It is observed that the maximum elongation was significantly higher in *A. pernyi* cocoon (L) (20.24 ± 1.9%) than *A. pernyi* cocoon (H) (14.29 ± 1.18%). Evidently, the maximum load of *A. pernyi* cocoon (L) (101.1 ± 10.62 N) specimens are much higher than those of *A. pernyi* cocoon (H) (125.79 ± 11.2 N). The results were confirmed by the relatively higher oriented fiber of the silk in *A. pernyi* (L). However, the tensile modulus of the *A. pernyi* cocoon (L) specimens in different directions is lower than that of the *A. pernyi* cocoon (H).

The mechanical properties of the cocoon, such as tensile modulus, maximum load, ultimate tensile strains, and ultimate tensile stress in different directions are summarized in Table 3. It can be seen that the maximum load and ultimate tensile stress of the *A. pernyi* cocoon (H) in the ±45° directions are evidently higher than those in the longitudinal and transverse directions. This is due to the anisotropic distribution of the silk orientations in the cocoon, resulting from the manner in which silkworm caterpillars spin silks [40]. This interesting phenomenon of anisotropic morphology may have essential meanings for the cocoons’ biological functions to protect pupae [41].

## 4. Conclusions

In summary, the microstructure and mechanical properties of A. pernyi cocoons from two different source regions were studied in this work. The obtained results are summarized below.

(1) There is a slightly different combination of morphology and properties that have adapted to coping with diverse local environments. In general, the *A. pernyi* (L) cocoon has a bigger size, heavier and thicker cocoon layer, and longer fiber bonding length than the corresponding part of *A. pernyi* (H).

(2) Both of these two *A. pernyi* cocoon composites share a similar fibre-network structure with fibroin fibers overlapped and connected by sericin binder. A large number of cubic crystals were attached to the surface of cocoon layer. The porosity of *A. pernyi* (H) cocoon layers are larger than the corresponding part of *A. pernyi* (L) cocoon layers.

(3) The protein secondary structure of the silk from the two regions is similar, while the absorption intensity of infrared light and vibration intensity are different.

(4) Tensile mechanical tests showed that the *A. pernyi* (L) cocoon behaved stronger and tougher. Besides, there are no significant differences among the results of the mechanical properties for 0°, ±45°, and 90° directions of these two cocoons.

(5) In general, *A. pernyi* (L) cocoons from northern China have relatively excellent performance compared to *A. pernyi* (H) cocoons, which is perhaps due to the physic-geographical environment and meteorological environment in northern China.

Understanding such natural composite structures and the mechanical behaviors will be the basis for the bionic design of new protective and light-weight fibrous materials and structures. Researchers pay attention to the design and develop next-generation bio-mimic protective materials on the basis of their biological functions, such as defense against natural enemies, thermal regulation, and anti-bacterial function.

## Figures and Tables

**Table 1:** Geometrical parameters of the *A. pernyi* cocoons from two different source regions (the error in this table is standard deviation).

	Weight (g)	Thickness (mm)	Length (mm)	Width (mm)		Ratio	
			2R_1_	2R_2_	2R_3_	R_1_/R_2_	R_1_/R_3_
*A. pernyi* (H)	0.54 ± 0.04	0.39 ± 0.08	41.45 ± 3.60	21.32 ± 1.43	22.51 ± 1.38	1.94	1.84
*A. pernyi* (L)	0.84 ± 0.05	0.45 ± 0.06	46.52 ± 3.10	23.89 ± 1.52	24.67 ± 1.55	1.95	1.89
*p*-value	1.14 × 10^−7^	7.30 × 10^−4^	2.11 × 10^−5^	4.98 × 10^−7^	2.11 × 10^−6^	-	-

**Table 2:** Fiber bonding length of different cocoon layers.

		Fiber Bonding Length (μm)	Porosity (%)
*A. pernyi* (H)	Outer layer	51 ± 9	25.65 ± 1.84
	Middle layer	61 ± 4	10.25 ± 1.02
	Inner layer	63 ± 5	5.21 ± 1.01
*A. pernyi* (L)	Outer layer	55 ± 4	11.62 ± 2.67
	Middle layer	65 ± 4	5.61 ± 0.66
	Inner layer	66 ± 5	3.70 ± 0.90

**Table 3:** The crystallization index of silk measured by infrared absorption spectroscopy (RA is relative absorbance).

	RA_1265_	RA_1235_	Crystallization Index
*A. pernyi* (H)	0.98	0.97	1.01
*A. pernyi* (L)	0.85	0.81	1.05

## Data Availability

The data presented in this study are available on request from the corresponding author.
